# The regulatory effect of homogeneous and spherical nanocrystal seeds on long-term retrogradation of corn starch

**DOI:** 10.1016/j.fochx.2026.104110

**Published:** 2026-06-16

**Authors:** Zijun Yu, Zhijie Zhu, Wenjing Li, Juan Tang, Mengxue Gui, Xu Chen, Manman Yu

**Affiliations:** Key Laboratory of Jianghuai Agricultural Product Fine Processing and Resource Utilization, Ministry of Agriculture and Rural Affairs, Anhui Engineering Research Center for High Value Utilization of Characteristic Agricultural Products, College of Food and Nutrition, Anhui Agricultural University, Hefei, China

**Keywords:** Corn starch, Retrogradation, Nanocrystal seeds, Recrystallization rate

## Abstract

The addition of nanocrystal seeds to promote retrogradation is a key strategy for producing resistant starch, However, the underlying mechanism remains unclear. In this study, spherical nanocrystal seeds (102 nm) were prepared through combined ethanol precipitation and acid hydrolysis, and their effects on the retrogradation of gelatinized corn starch (CS) were investigated. During storage, the crystallinity of CS increased from 1.74% at Day 0 to 6.59% at Day 28. For samples with added nanocrystal seeds, CS crystallinity increased from 1.78% to 8.25%, indicating that nanocrystal seeds facilitated the development of long-range ordered structures. Notably, the addition of nanocrystal seeds also increased the starch recrystallization rate constant from 0.25 to 0.39, confirming accelerated starch retrogradation. Moreover, nanocrystal seeds promoted the formation of a cross-linked network morphology and improved the thermal stability of retrograded starch. In summary, these findings provide mechanistic insights into the role of nanocrystal seeds in accelerating starch retrogradation.

## 1 Introduction

Starch retrogradation is a physicochemical process in which gelatinized starch molecules rearrange from an unordered, amorphous state to an ordered, crystalline structure during cooling or storage (Liu et al., 2024). This process is typically divided into two stages: short-term retrogradation and long-term retrogradation (Zhang et al., 2011). Short-term retrogradation mainly involves rapid rearrangement and crystallization of amylose molecules, and determines the initial texture of food, whereas long-term retrogradation mainly involves slow recrystallization of amylopectin side chains, and determines the long-term storage stability and deterioration of food—for example, hardening and retrogradation (Tao et al., 2024). The retrogradation behavior of starch is profoundly influenced by various factors, including the ratio of amylose/amylopectin, storage temperature, moisture content, and the presence of other components (e.g. lipids and proteins) in the food system (Liu et al., 2021; Ning et al., 2025).

From the perspective of crystallization kinetics, starch retrogradation process is essentially a recrystallization process that typically involves three core stages: nucleation, crystal growth, and perfect crystal formation (Oh et al., 2017; Zheng & Zeng, 2025). Among these stages, nucleation is the initial step and plays a key role in determining the overall recrystallisation rate. Based on this, researchers have proposed the addition of exogenous crystal seeds to provide ready-made nucleation sites for starch molecules, thereby effectively inducing and accelerating the formation of crystalline structures and enabling precise control of the recrystallization process (Zhu et al., 2020). Among the various types of crystal seeds, nanocrystal seeds have attracted considerable attention due to their unique size-related advantages. Compared with conventional micrometer-sized crystal seeds, nanocrystal seeds have a larger specific surface area (SSA) and more nucleation sites, which increase their tendency to aggregate (Kim et al., 2013). Their small particle size also enables more uniform dispersion in the starch matrix, promoting the adsorption and ordered arrangement of starch molecule chains on their surfaces and significantly accelerating the recrystallization rate.

Acid hydrolysis is widely used for preparing nanocrystal seeds because of its rapid and efficient processing characteristics (Chen et al., 2022; Qin et al., 2016). However, the resulting nanocrystal seeds often exhibit heterogeneous particle sizes and a small SSA, which limit their effectiveness in promoting starch retrogradation. These limitations likely arise from the high molecular weight and broad chain length distribution of the starch prior to acid hydrolysis. To address these limitations, ethanol precipitation can be used to fractionate starch chains on the basis of their differential solubility at varying ethanol concentrations. Precise adjustment of the ethanol concentration enables selective precipitation of a low-molecular-weight linear dextrin fraction with a narrow chain length distribution (Chang et al., 2018; Wang et al., 2019). Based on this, the present study proposes a core hypothesis: using the low-molecular-weight dextrin obtained through high-concentration ethanol precipitation could serve as a precursor for the preparation of large-SSA nanocrystal seeds through controlled acid hydrolysis. Incorporating these nanocrystal seeds into gelatinized corn starch would significantly promote its long-term retrogradation.

Accordingly, this study systematically describes the preparation of nanocrystal seeds and reports the effects of nanocrystal seeds addition on the long-term retrogradation of corn starch. A series of analytical techniques, such as atomic force microscopy (AFM), laser particle size analyzer, X-ray diffraction (XRD), Fourier transform infrared spectroscopy (FTIR), differential scanning calorimetry (DSC), scanning electron microscope (SEM), and thermogravimetric analysis (TGA), were used to comprehensively characterize the multi-scale structural changes in starch samples during retrogradation. This study aims to provide a more detailed theoretical basis for the use of nanocrystal seeds to promote long-term starch retrogradation, thereby supporting industrial-scale production of retrograded starch with high resistant starch content.

## 2 Materials and methods

### 2.1 Materials

Corn starch (S11149 amylose content: 27.81%) was purchased from Shanghai Yuanye Biotechnology (Shanghai, China). High-amylose maize starch (amylose content: 60.74% ± 2.14%) was purchased from Henan Hengrui Starch Technology Co., Ltd. (Henan, China). Ethanol (G73537B) was purchased from Shanghai Titan Technology (Shanghai, China). pullulanase (CAS 9075-68-7, ≥ 1000 NPUN/g) was purchased from Shanghai Aladdin Biochemical Technology Co., LTD. (Shanghai, China). All the other reagents used were of analytical grade.

### 2.2 Preparation of nanocrystal seeds

The preparation of nanocrystalline seeds was based on previous research with slightly modified (Jia et al., 2023; Zhu et al., 2020). A 10% high-amylose maize starch suspension was fully gelatinized at 140 °C in a reaction vessel for 40 min. Then the mixture was cooled to 60 °C, and pullulanase (CAS 9075-68-7, ≥ 1000 NPUN/g) purchased from Shanghai Aladdin Biochemical Technology Co., Ltd. was added at a dosage of 10% (on a dry starch basis). Enzymatic hydrolysis was then carried out at 60 °C for 6 h. After a complete reaction, the solution was placed in a boiling water bath for 20 min to inactivate the enzymes. The resulting mixture was centrifuged at 5000 ×*g* for 10 min, and the supernatant was sequentially added with ethanol at varying concentrations (30%, 40%, 50%, 60% and 70%) for centrifugal precipitation to obtain linear dextrin of different degrees of polymerization (DP). Based on our previous research (Yu et al., 2026), the linear dextrin with good crystalline structure, obtained by precipitation at 70% ethanol concentration, were collected and freeze-dried. The dried linear dextrin was subsequently prepared as a 10% suspension using 3.16 mol/L sulfuric acid and subjected to acid hydrolysis at 45 °C for 7 d. After acid hydrolysis, the samples were washed with anhydrous ethanol until neutral and dried at 40 °C for 12 h and stored for later use. The control group was established using linear dextrin that had not undergone acid hydrolysis.

### 2.3 Preparation of retrograded starch

The sample preparation procedure was slightly modified from previous studies (Chi et al., 2022). Specifically, 3 g of corn starch was added to deionized water to prepare a 5% (*w*/w) starch suspension. The mixture was fully gelatinized by heating at 95 °C for 30 min, then transferred to a 40 °C water bath and stirred until the temperature decreased to 45 °C. Subsequently, nanocrystal seeds equivalent to 7% of the starch dry weight were added and stirred at 45 °C for 10 min until complete dissolution, following the method described by Wang et al. (2020). The prepared samples were cooled to room temperature and stored sealed for 0 d, 1 d, 2 d, 7 d, 14 d, and 28 d at 4 °C, respectively, followed by freeze-drying. During this process, visual inspection and odor testing ensured that the samples were not contaminated by microorganisms. These samples were designated as J-CS-0d, J-CS-1d, J-CS-2d, J-CS-7d, J-CS-14d and J-CS-28d. As a control, gelatinized corn starch was retrograded for the same time, which was also freeze-dried and labeled correspondingly as CS-0d, CS-1d, CS-2d, CS-7d, CS-14d, and CS-28d.

### 2.4 Atomic force microscope (AFM)

The surface structure of the starch particles after modification was characterized using the AFM instrument (Dimension ICON, Bruker Inc., Germany). Firstly, the sample was dispersed in anhydrous ethanol and then dispersed on a mica sheet after being ultrasonicated for 10 min. The sample was scanned in the air at room temperature, with a resonance frequency of 70 kHz and a spring constant of 0.4 N/m. The surface morphology and surface characteristics information were obtained by the interaction force between the tip on the microcantilever and the sample surface.

### 2.5 Particle size distribution

The particle size distribution of the sample was determined using a laser particle size analyzer (Mastersizer 2000, Malvern Instruments, UK). First, the starch sample was added to deionized water and then stirred continuously to disperse the particles in the solution. The particle size distribution was then recorded directly using the instrument's accompanying software.

### 2.6 X-ray diffraction (XRD)

The samples were first sieved through a 200-mesh screen to ensure uniform particle size. Their crystalline structures were then characterized using a SmartLab SE X-ray diffractometer (Rigaku, Japan) with Cu Kα radiation. Data were collected in the 2θ range of 5°–50° at a scanning rate of 5°/min. The relative crystallinity was determined using MDI Jade 6.0 (Materials Data, Inc., USA) software, calculated according to the following formula (Chen et al., 2017):

$$\mathrm{Relative crystallinity}\;\left(\%\right)=\frac{I_{c}}{I_{a}+I_{c}}\times 100\%$$

Where *Ic* and *Ia* represented the crystalline region area and amorphous region area, respectively.

### 2.7 Fourier transform infrared spectroscopy (FTIR)

The infrared spectrum of the sample was acquired using a Fourier transform infrared spectrometer (IS50, Thermo Nicolet Corporation, USA) over a scanning range of 4000–400 cm^−1^. Specifically, 1 mg of the sample was accurately weighed and mixed with 100 mg of potassium bromide (KBr) in a mortar. After thorough grinding, then compressed it into a thin sheet for spectroscopic analysis. All spectra were processed by deconvolution using OMNIC 9.2 (Thermo Fisher Scientific, Inc., USA) software, and the absorbance ratio at 1047 cm^−1^ to 1022 cm^−1^ was evaluated to assess the short-range orderliness. The hydrogen bond energy (*E*) was calculated using the following formula (Cui et al., 2025):

$$E=\frac{1}{k}\times \frac{v_{0}-v}{v_{0}}$$

where *ν*_*0*_ is the standard frequency corresponding to free O—H groups (3650 cm^−1^), *ν* is the wavenumber of the O—H groups of starch, and *k* is a constant (1/*k* = 262.5 kJ).

### 2.8 Differential scanning calorimetry (DSC)

The thermal characteristics of the samples were analyzed using a differential scanning calorimeter (DSC8000, PerkinElmer, USA). A sample aliquot of 2 mg along with 6 μL of deionized water was precisely weighed into a liquid crucible and equilibrated at 25 °C for 24 h. The DSC scan was performed from 30 °C to 110 °C at a heating rate of 10 °C/min to collect enthalpy data for Avrami theory analysis. All experiments were carried out in triplicate. The Avrami equation is commonly used to analyze isothermal crystallization processes and has been widely applied in studies of starch retrogradation. Therefore, this equation is employed to describe the kinetics of starch retrogradation (Xu et al., 2012):

$$X\left(t\right)=\frac{{\mathrm{\Delta H}}_{t}-{\mathrm{\Delta H}}_{0}}{{\mathrm{\Delta H}}_{\infty }-{\mathrm{\Delta H}}_{0}}=1-\exp\left(-kt^{n}\right)$$

where *X(t)* represents the fraction of starch crystallized at time t; *ΔH*_*0*_*, ΔH*_*t*_, and *ΔH*_*∞*_ are the enthalpy changes at 0 h, t h, and 24 h, respectively; *n* is the Avrami exponent; and *k* is the crystallization rate constant. Enthalpy changes are expressed in joules per gram of dry starch (J/g).

### 2.9 Scanning electron microscopy (SEM)

The starch sample was evenly distributed on the conductive stage and subsequently coated with a thin gold film via vacuum sputtering for 60 s. Surface morphology of the starch particles was examined using a scanning electron microscope (Hitachi, S-4800, Japan) at an accelerating voltage of 3.0 kV. Images were acquired at various magnifications (500× and 1000×) to reveal microstructural details.

### 2.10 Thermogravimetric analysis (TGA)

The thermal stability of the samples was assessed by thermogravimetric analysis using a Discovery TGA55 instrument (TA Instruments, USA). 3.0 mg of sample was precisely weighed and loaded into a solid crucible. The measurement was conducted under a nitrogen with a flow rate of 60 mL/min, while the temperature was increased from 30 °C to 600 °C at a heating rate of 10 °C/min in ramp mode. The temperature corresponding to the maximum rate of weight loss was determined using the built-in TRIOS software of the instrument.

### 2.11 Statistical analysis

All experiments were independently repeated at least three times. The results were expressed as the mean ± standard deviation (SD). Correlation analysis was performed using IBM SPSS Statistics version 27 software to assess significant differences at *p*-value ≤0.05. The graphs were accomplished using Origin 2021 software (OriginLab Corporation, USA).

## 3 Results and discussion

### 3.1 AFM results and size distribution of the nanocrystal seeds

**Fig. 1:**
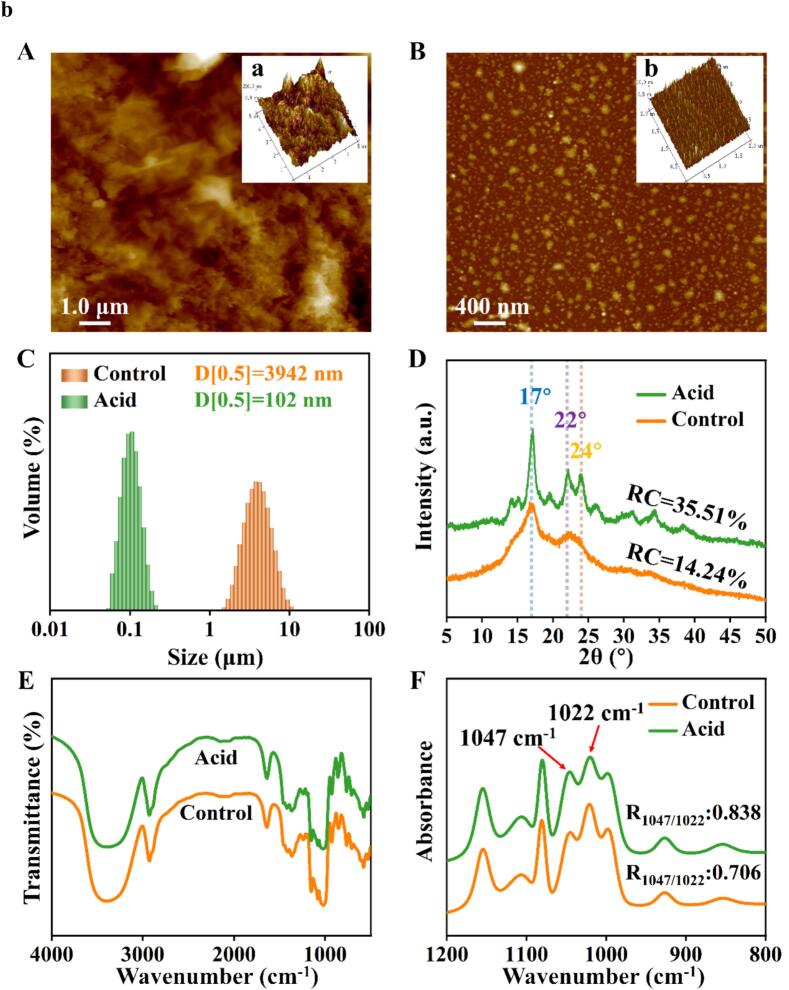
The two-dimensional and three-dimensional topographic images of atomic force microscopy before (A and a) and after (B and b) acid hydrolysis. Particle size distribution (C), X-ray diffractograms (D), Fourier transform infrared spectra curves (E) and deconvoluted spectra curves (F) before and after acid hydrolysis.

AFM was used to investigate the morphology of nanocrystal seeds in this study. Two-dimensional (2D) and three-dimensional (3D) topographic images of the nanocrystal seeds are presented in Fig. 1A and B. The control sample had relatively larger sizes and highly fluctuating morphologies with pronounced differences in the height values on their surfaces. The topographic images indicated that the control samples were heterogeneous on the micrometer scale. By contrast, the acid hydrolysis samples exhibited a distinct spherical granular morphology with relatively small particle sizes. Furthermore, these samples demonstrated greater homogeneity in both vertical and horizontal directions. The change in topographic morphology may be attributable to preferential hydrolysis of the loose amorphous and poorly crystalline regions by acid treatment, leaving behind more acid-resistant crystalline regions and inducing the formation of smaller starch particles (Wang & Copeland, 2015). This finding is consistent with those of relevant studies, in which acid hydrolysis disrupted the original crystalline structure of starch and led to the formation of more acid-resistant and homogeneously distributed nanoparticles (Gonzalez & Wang, 2023; Wang et al., 2012).

The size distribution of the nanocrystal seeds was further analyzed using a laser particle size analyzer, and the results are presented in Fig. 1C. The control group had a relatively large average particle size (3942 nm), whereas the acid hydrolysis sample exhibited a significantly smaller particle size distribution, with an average particle size of 102 nm. Notably, the acid hydrolysis sample exhibited greater homogeneity (uniformity = 0.33) than did the control group (uniformity = 0.23), and had smaller average surface and volume particle sizes (Table S1). These results further confirmed that acid hydrolysis was an effective treatment method for preparing homogeneous nanocrystal seeds by selectively breaking down its loose amorphous regions and poorly crystalline structure.

### 3.2 Crystalline structure of nanocrystal seeds

Fig. 1D depicts the XRD patterns of the linear dextrin before and after acid hydrolysis. Both samples exhibited distinct B-type crystalline characteristic peaks at 17°, 22°, and 24°, indicating that acid hydrolysis did not alter the crystalline type of starch. Notably, the relative crystallinity of linear dextrin increased from 14.24% to 35.51% after acid hydrolysis. This increase may be because acid hydrolysis preferentially damaged the amorphous and poorly crystalline regions of the linear dextrin, which released unbound starch chains. Consequently, these released starch chains then spontaneously reassociated and assembled into a crystalline structure with higher crystallinity (Li et al., 2024; Qiao et al., 2017).

FTIR is an important technique for characterizing the structure of organic compounds based on molecular vibrations, which was used to investigate the ordered helical structures (short-range ordered structures) of the nanocrystal seeds (Pan et al., 2026). As shown in Fig. 1E, acid hydrolysis did not generate any new characteristic functional group peaks, indicating that this treatment did not change the functional group composition of the original starch. In order to further quantify the short-range ordered structure of the nanocrystalline seeds, the FTIR spectra were deconvoluted. Due to the absorption bands at 1047 cm^−1^ and 1022 cm^−1^ corresponding to crystalline and amorphous structures, respectively, the intensity ratio of these bands (R_1047/1022_) was used as a quantitative measure of the short-range orderliness in the crystalline structure of starch. A higher R_1047/1022_ value indicates a greater degree of short-range orderliness (Liu et al., 2021). It was observed that the R_1047/1022_ value increased from 0.706 to 0.838 after acid hydrolysis (Fig. 1F), reflecting an enhancement in the short-range orderliness of the starch crystalline structure. The improvement in short-range ordered structure further confirmed that acid hydrolysis can improve the structural orderliness of the linear dextrin, which was consistent with the XRD results. In summary, acid hydrolysis treatment can produce nanocrystal seeds with a highly ordered structure by destroying the amorphous and poorly crystalline regions within the linear dextrin.

### 3.3 XRD patterns of retrograded starch

**Fig. 2:**
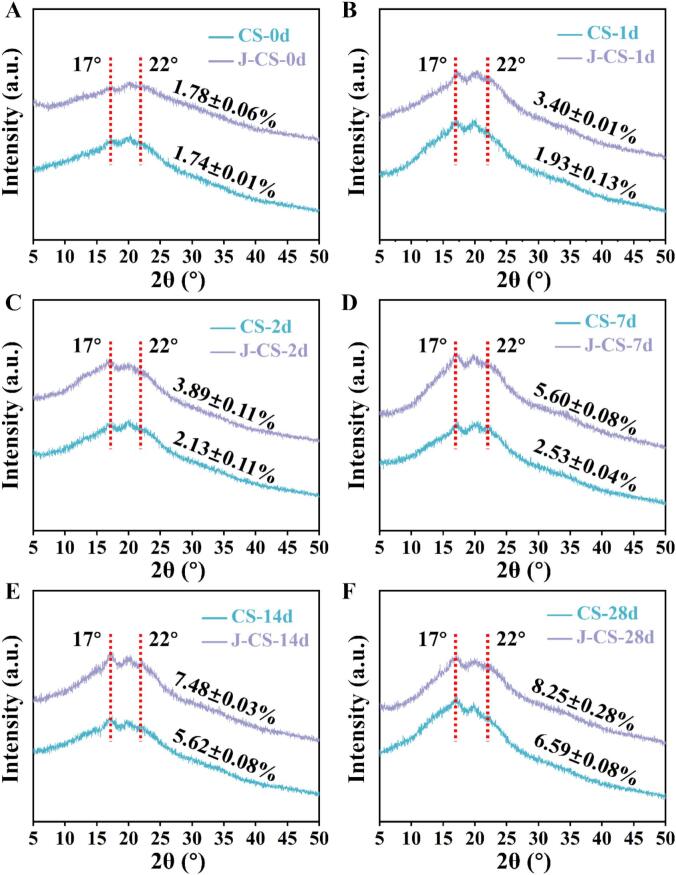
X-ray diffractograms of CS samples and J-CS samples during 28-day retrogradation.

Fig. 2 presents the XRD patterns of the samples with or without nanocrystal seeds during a 28-d retrogradation time. It can be observed that all samples have distinct characteristic peaks at 17°, 20°, and 22°, indicating that the crystalline structure was B + *V*-type crystals. The B-type characteristic peak was caused by the recrystallization of starch resulting from the association of hydrogen bond during retrogradation. By contrast, the V-type characteristic peak represented by 20° was formed because of the hydrophobic interaction between starch and endogenous lipids (Ye et al., 2018). At 0 d, the CS and J-CS groups showed similar relative crystallinity values (1.74% and 1.78%, respectively), with no significant differences between them, confirming that the addition of nanocrystal seeds did not affect the initial crystallinity of the system. Notably, the J-CS samples exhibited higher crystallinity than did the CS samples over the same retrogradation period (1–28 d). Particularly, the J-CS samples reached a much higher growth rate (363%) than the CS samples (279%) after 28 d storage, where the ‘growth rate’ refers to the percentage increase relative to the initial value at 0 d, calculated as [(value at 28 d – value at 0 d) / value at 0 d] × 100%. This might be because during the retrogradation process of starch, the addition of nanocrystal seeds can serve as the initial crystal nucleus, shortening the time for starch chains to self-assemble into a nucleus, thereby accelerating the formation of an ordered crystalline structure. This finding is consistent with previous studies, which have shown that the addition of nanocrystal seeds promotes starch retrogradation (Wang et al., 2020).

### 3.4 FTIR spectra of retrograded starch

**Fig. 3:**
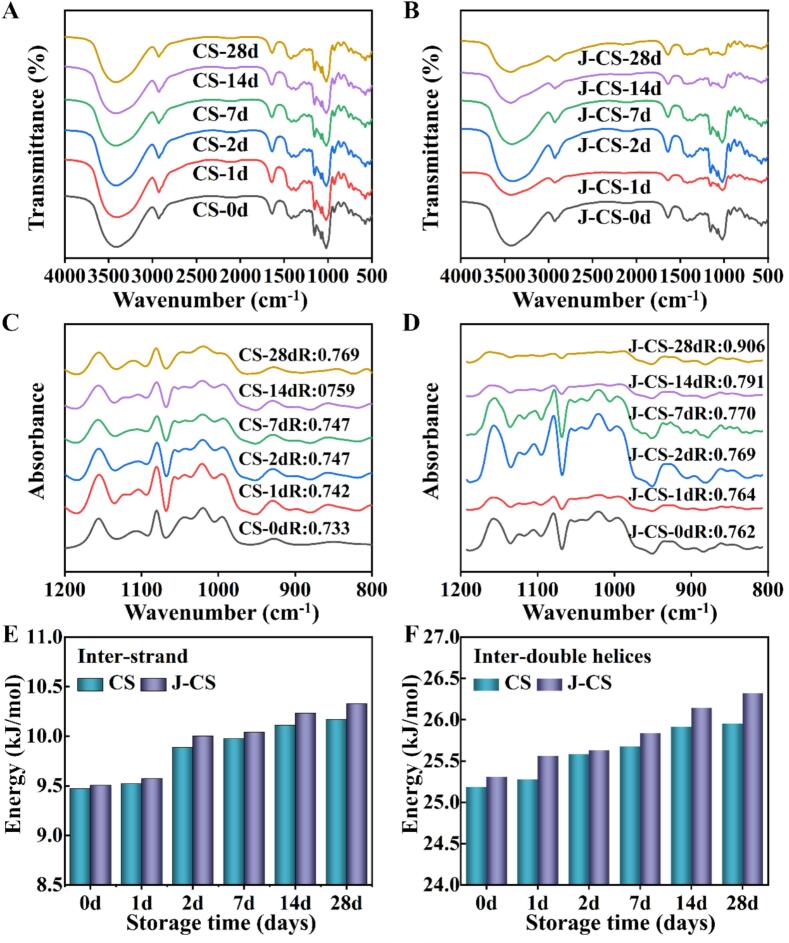
Fourier transform infrared spectra curves (A and B), deconvoluted spectra curves (C and D), inter-strand hydrogen bond (E) and inter-double helices hydrogen bond (F) of CS samples and J-CS samples during 28-day retrogradation.

FTIR spectra of the CS and J-CS samples during storage are presented in Fig. 3A and B. It can be observed that the spectral curves of all the samples showed the similar peak shape, with no appearance of new peaks or disappearance of existing peaks. This indicated that the addition of exogenous nanocrystal seeds did not change the functional group composition of the retrograded starch. The spectra obtained after deconvolution of the original FTIR spectra are presented in Fig. 3C and D. At 0 d, the R_1047/1022_ values of the CS and J-CS samples were 0.73 and 0.76, respectively. As retrogradation progressed to 28 days, the R_1047/1022_ value for the CS-28d sample gradually increased to 0.77, corresponding to an increase of 4.91% relative to that of the CS-0d sample (Fig. 3C). This increase may be attributable to the formation of double-helical structures through the formation of hydrogen bonds between starch chains during retrogradation, which enhanced the short-range orderliness of the CS samples. In comparison, the R_1047/1022_ value of the J-CS samples gradually increased to 0.91, corresponding to an increase rate of 18.90% (Fig. 3D). Notably, the increase rate of J-CS samples was 3.91-fold higher than that of the CS samples after retrograding for 28 d. This may be attributed to the addition of the nanocrystal seeds, which facilitated the association of starch chains, and thereby promoted the formation of more ordered short-range structure.

The improvement in the short-range orderliness of retrograded starch can be further evaluated by measuring the strength of hydrogen bonds. According to previous reports, the characteristic positions of the inter-strand hydrogen bond and inter-double helix hydrogen bond were approximately 3511 cm^−1^ and 3290 cm^−1^, respectively (Lu et al., 2021). The calculated hydrogen bond energy values for inter-strand and inter-double-helix interactions in the retrograded starch samples are presented in Fig. 3E and F. At 0 d, the inter-strand hydrogen bond energy and inter-double helix hydrogen bond energy of the CS samples were 9.47 kJ/mol and 25.19 kJ/mol, respectively. As the retrogradation time extended to 28 d, these values gradually increased to 10.17 kJ/mol and 25.96 kJ/mol, respectively (Fig. 3E). This trend was likely due to the gradual association of starch chains during the retrogradation process, leading to the formation and stabilisation of double-helical structures through hydrogen bonding. Moreover, the degree of this association gradually deepened as the retrogradation time increased, resulting in the bond energies between inter-strand and inter-double helix hydrogen bonds gradually increasing. Importantly, after 28 d of retrogradation, the inter-strand and inter-double helix hydrogen bond energies of the J-CS samples increased from the initial 9.51 kJ/mol and 25.31 kJ/mol to 10.33 kJ/mol and 26.32 kJ/mol, respectively (Fig. 3F). The corresponding increase rates for the inter-strand and inter-double helix hydrogen bond energies in the J-CS samples were 1.17- and 1.31-fold than those of the CS samples. This difference may be because the introduction of nanocrystal seeds provided the necessary nuclei for the formation of crystalline structure during starch retrogradation. Thus, the starch chains could rapidly attach to the surface of the nanocrystal seeds and arrange in an orderly manner, which in turn significantly promoted the formation of both inter-strand and inter-double helix hydrogen bond formation. In summary, the added nanocrystal seeds served as the crystal nucleus that facilitated hydrogen bond formation and enhanced the short-range order of the retrograded starch.

### 3.5 DSC results of retrograded starch

**Fig. 4:**
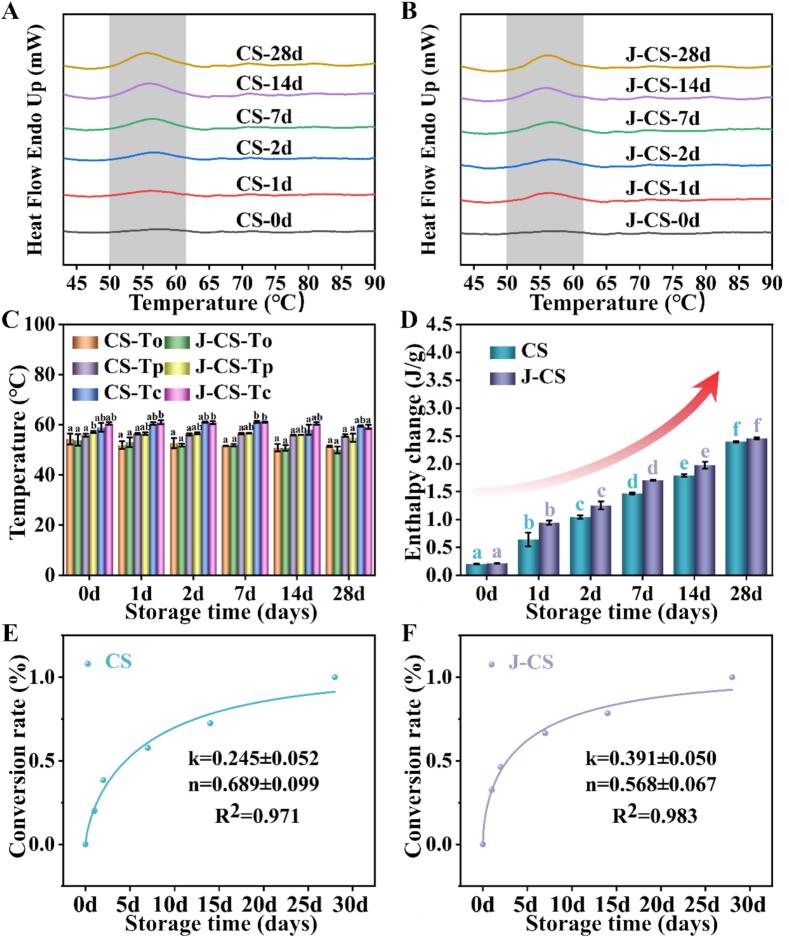
Differential scanning calorimetry curves (A and B), the value of To Tp and Tc (C), enthalpy change values (D) and retrogradation kinetics curves (E and F) of WS samples and WS-LA samples during 28-day retrogradation.

The thermal parameters obtained from DSC could reflect the crystalline characteristics of the retrograded starch, such as the degree of crystalline perfection, crystal morphology, crystal size, and structural uniformity (Warren et al., 2016). Fig. 4A and B presented the DSC curves of corn starch with or without nanocrystal seeds at different retrogradation time points. An endothermic peak was observed at approximately 50–60 °C in both the CS and J-CS samples (Fig. 4C), and the intensity of this peak both progressively increased with increasing retrogradation time. This increase may be attributable to the formation of more stable double-helical structures by starch chains during storage, which required greater energy for disruption. In addition, the change in enthalpy (ΔH) can quantify the energy required to disrupt the double helix structure and eliminating the crystalline structure of starch. At 0 d, the ΔH values of the CS and J-CS samples were 0.20 J/g and 0.21 J/g, respectively. As the retrogradation time increased to 28 d, the ΔH value of the CS samples gradually increased to 2.40 J/g, corresponding to a net increase of 2.20 J/g. Prominently, the enthalpy value of J-CS samples increased to 2.46 J/g, with a net increase of 2.25 J/g (Fig. 4D). At each retrogradation time point, the J-CS samples exhibited higher enthalpy values than did the CS samples. These results indicated that the addition of nanocrystal seeds promoted the formation of double helix structures and enhanced the thermal stability of retrograded starch.

In order to better characterize the dynamic process of starch retrogradation, we used the Avrami model to analyze the recrystallization kinetics of retrograde starch (Wang et al., 2021). The recrystallization rate constant (k) depended on the concentration of crystal nuclei, and a higher k value indicates the formation of a greater number of nuclei during starch retrogradation. Notably, the k value of J-CS samples (0.391) was higher than that of the CS samples (0.245). Therefore, the J-CS samples exhibited a faster recrystallisation rate than did the CS samples. In addition, the Avrami index (n) depends on the nucleation type and crystal growth dimensions rather than the number of nuclei (Chi et al., 2022). Fig. 4E and F showed the recrystallization kinetics curves of the CS samples and the J-CS samples at different retrogradation time points. It can be observed that the n values of both sample groups were less than 1, indicating that the main nucleation type has not changed after the addition of the nanocrystal seed, and the nucleation type was still instantaneous. A higher n value was usually associated with a slower rate of retrogradation (Shi & Gao, 2016). By comparing, it can be found that the n value of the J-CS samples (0.568) was lower than that of the CS samples (0.689), indicating that the addition of the nanocrystal seeds accelerated the retrogradation process. In conclusion, these results demonstrated that nanocrystal seeds addition significantly promoted the recrystallization process of corn starch.

### 3.6 Micromorphology analysis of retrograded starch

**Fig. 5:**
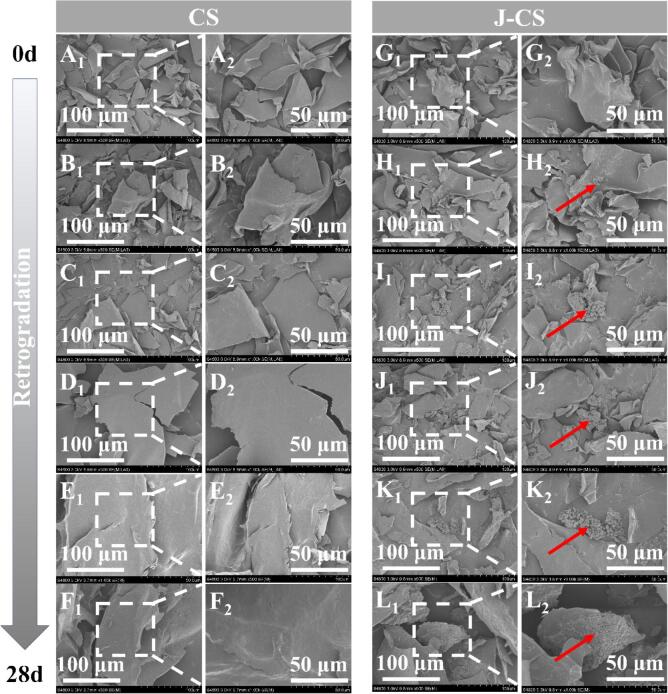
Scanning electronic microscope of WS samples (A-F) and WS-LA samples (G-L) during 28-day retrogradation.

Starch retrogradation is often accompanied by significant changes in its microtopography. Fig. 5 shows the scanning electron microscopy images of retrograded starch with or without nanocrystal seeds during storage. It can be observed that as the retrogradation time increased from 0 d to 28 d, the CS samples gradually transformed from an initially fragmented structure into a relatively dense, sheet-like morphology (Fig. 5A–5F). This observation is consistent with that of a previous study reporting that the retrogradation process of chestnut starch retrogradation involved morphological transition from irregular fragments to tightly aggregated sheet-like structures (Liu et al., 2024). By contrast, the J-CS sample displayed similar fragmented characteristics at 0 d (Fig. 5G). However, with increasing retrogradation time, the morphology of the J-CS samples showed distinct granules cross-link, especially at 28 d, the cross-linking phenomenon became particularly pronounced (Fig. S1). This behavior might be because the addition of nanocrystal seeds provided nuclei for the formation of the crystalline structure, the starch chains could attach to the surfaces of these nuclei and continuously bond and grow during the retrogradation process, thereby leading to the occurrence of this cross-linking phenomenon.

### 3.7 TGA results of retrograded starch

**Fig. 6:**
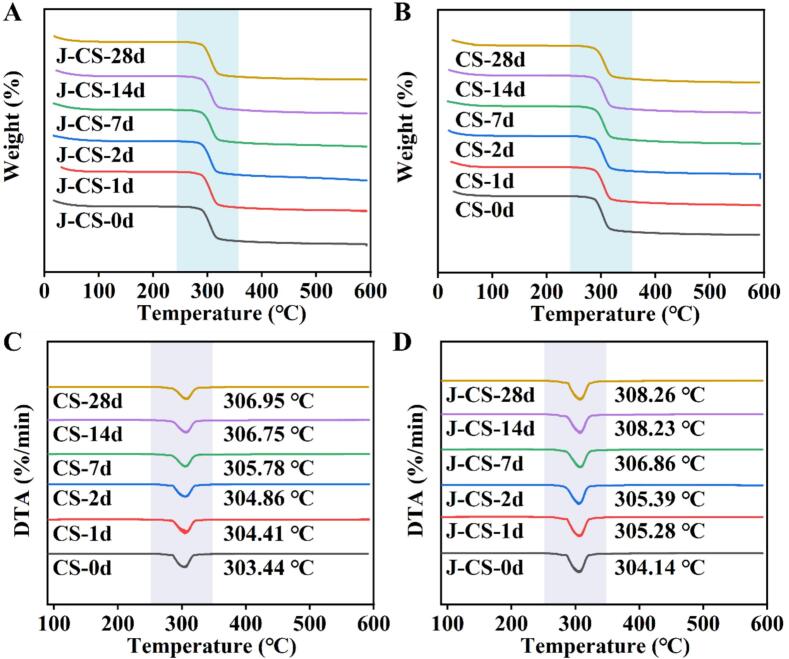
Thermogravimetric curves (A and B) and differential thermogravimetric curves (C and D) of CS samples and J-CS samples during 28-day retrogradation.

TGA and derivative thermogravimetry were used to evaluate the thermal stability of retrograded starch, as illustrated in Fig. 6. All the samples exhibited two stages of weight loss, with the first stage (30–150 °C) representing the evaporation of free water in the starch, and the second stage (150–600 °C) corresponding to the degradation of starch molecules (Sun et al., 2025). It can be observed that the maximum weight loss for all samples occurred in the second stage of thermal degradation (Fig. 6A and B). The temperatures corresponding to the maximum weight loss of the CS and J-CS samples were 303.44 °C and 304.14 °C at 0 d, respectively. As the retrogradation time increased to 28 d, the maximum weight loss temperature of the CS sample increased to 306.95 °C, representing an increase of only 1.16%. In contrast, the values of the J-CS sample exhibited a greater increase to 308.26 °C, corresponding to a 1.35% rise (Fig. 6C and D). This difference may be attributed to the fact that the addition of nanocrystal seeds promoted a further increase in starch crystallinity after 28 d of retrogradation (as supported by the XRD results), thereby enhancing the thermal stability of the starch. In summary, the addition of nanocrystal seeds promoted the retrogradation of starch and increased the thermal stability of retrograded starch.

### 3.8 General discussion

**Fig. 7:**
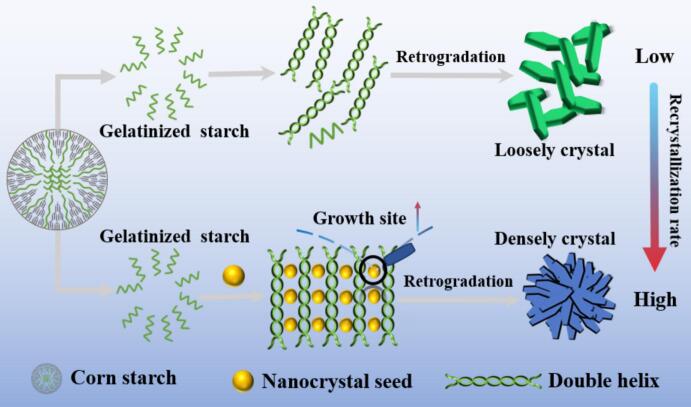
Schematic diagram for the conformational transition promoting starch retrogradation by nanocrystal seeds.

The crystallization of starch during retrogradation typically involves three stages: nucleation, crystal growth, and the eventual formation of a well-ordered crystalline structure (Chang et al., 2021; Zhang et al., 2025). Accordingly, this study systematically investigates whether the introduction of exogenous nanocrystal seeds can actively accelerate the retrogradation of corn starch. The schematic diagram of this potential mechanism is shown in Fig. 7. After gelatinization, the native crystalline structure of corn starch was completely disrupted, and a fraction of the amylose may reorganize into a loose crystalline structure during subsequent cooling or drying. With the retrogradation time increased, the double helix structures gradually formed and further assembled into relatively loose B-type crystalline structure. By contrast, the addition of exogenous nanocrystal seeds can serve as the necessary nucleus for forming the crystalline structure of starch. The Avrami index results further support this nucleation role: the nanocrystal seeds significantly shorten the induction period and increase the crystallization rate constant, which cannot be explained by a simple physical addition. The starch chains attached to the surface of the nanocrystal seeds cross-link with each other and grow rapidly, eventually resulting in a more compact crystalline structure. However, the absence of preservatives during sample preparation is one of the limitations of this study. Future research can introduce antibacterial agents or more rigorous aseptic testing to ensure the reliability of the experimental results. In summary, compared with gelatinized corn starch, the recrystallization rate of starch was significantly increased after adding the nanocrystal seeds, leading to the formation of a more ordered crystalline structure. These results demonstrated that exogenous nanocrystal seeds can effectively promote starch retrogradation, offering new perspectives for the rapid production of retrograded starch with high crystallinity, which can be directly translated into industrial practice by adding nanocrystal seeds during retrogradation to shorten processing time for producing resistant starch used in low-glycemic foods.

## 4 Conclusion

This study confirmed that the addition of homogeneous spherical nanocrystal seeds (102 nm) effectively accelerated the long-term retrogradation of gelatinized corn starch. Compared with the control groups, in which crystallinity increased from 1.74% to 6.59% over the storage period, the samples containing nanocrystal seeds exhibited a more pronounced rise from 1.78% to 8.25%. Consistent with this structural enhancement, these samples with adding nanocrystal seeds also demonstrated a higher degree of short-range order and developed a more extensive hydrogen-bond network throughout during retrogradation than did the control samples. Particularly, Avrami model kinetics analysis revealed that the addition of nanocrystal seeds increased the recrystallization rate constant from 0.25 to 0.39, providing direct kinetic evidence for their role in accelerating starch retrogradation. Furthermore, the nanocrystal seeds promoted the formation of a cross-linked network morphology and enhanced the thermal stability of the retrograded starch. Collectively, these findings not only highlight the use of tailored nanocrystal seeds as an effective strategy for producing highly crystalline retrograded starch but also provide deeper mechanistic insights into how nanocrystal seeds accelerate starch retrogradation.

## CRediT authorship contribution statement

**Zijun Yu:** Writing – review & editing, Writing – original draft, Methodology, Formal analysis, Data curation. **Zhijie Zhu:** Writing – review & editing, Methodology, Formal analysis. **Wenjing Li:** Validation, Methodology. **Juan Tang:** Methodology, Formal analysis. **Mengxue Gui:** Methodology. **Xu Chen:** Writing – review & editing, Supervision, Funding acquisition, Formal analysis, Conceptualization. **Manman Yu:** Writing – review & editing, Validation, Methodology.

## Declaration of competing interest

The authors declare that they have no known competing financial interests or personal relationships that could have appeared to influence the work reported in this paper.

## Data Availability

Data will be made available on request.
