# Supplementary material for: The regulatory effect of homogeneous and spherical nanocrystal seeds on long-term retrogradation of corn starch
Source: Food Chem X. 2026 Jun 16;37:104110. doi: 10.1016/j.fochx.2026.104110 (PMC13312491; doi:10.1016/j.fochx.2026.104110)
Supplement: Supplementary material [file mmc1.docx]

**Table. S1** The particle size distribution results before and after acid hydrolysis.

|  | D [3,2] (nm) | D [4.3] (nm) | D [0.1] (nm) | D [0.5] (nm) | D [0.9] (nm) | Uniformity |
| --- | --- | --- | --- | --- | --- | --- |
| Control | 0.099±0.00 | 0.107±0.00 | 0.072±0.00 | 0.102±0.00 | 0.148±0.00 | 0.234±0.00 |
| Acid | 3.671±0.08 | 4.25±0.09 | 2.384±0.05 | 3.942±0.10 | 6.546±0.08 | 0.330±0.00 |


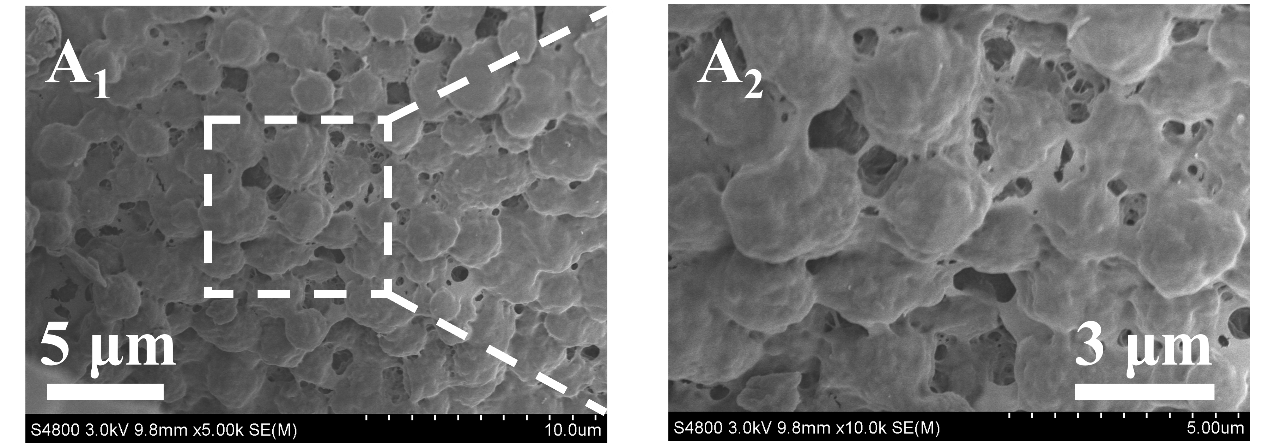


**Fig. S1** Scanning electron microscope image of corn starch after retrogradation for 28 days with the addition of nanocrystal seeds.
